# Delegates from Georgia to the Pan-American Medical Congress

**Published:** 1893-09

**Authors:** 


### DELEGATES FROM GEORGIA TO THE PAN-AMERICAN MEDICAL CONGRESS.

Governor Northen has appointed the following delegates from Georgia to the Pan-American Medical Congress to be in session at Washington, D. C., September 5th-8th : Drs. W. O'Daniel, W. P. Nicolson, J. S. Todd, W. S. Kendrick, A. W. Calhoun, Dan. H. Howell, Robert Battey, J. B. S. Holmes, C. P. Gordon, W. B. Tate, K. P. Moore, C. H. Hall, J. C. LeHardy, R. J. Nunn, J. W. Duncan, W. H. Elliott, Eugene Foster, DeSaussure Ford, W. H. Doughty, J. A. Dunwoody, G. J. Grimes, Dr. Seth Jordan, I. H. Goss, S. C. Benedict, A. A. Smith, N. J. Jelks, G. T. Miller, J. I. Darby, Reuben Nesbit, Lawrence Felder, H. Holtzclaw, J. G. Hopkins, T. M. McIntosh, A. M. Burt, J. W. Bailey, J. H. Malone, P. R. Cortelyou, Dr. Tennant, H. Perdue, F. M. Ridley, W. H. Philpot, E. L. Bardwell, J. M. Kelly, R. H. Taylor, W. C. Kendrick and N. L. Long.

